# Enzyme-linked immunosorbent assay for diagnosis of *Amphimerus* spp. liver fluke infection in Humans

**DOI:** 10.1590/0074-02760160426

**Published:** 2017-03-27

**Authors:** William Cevallos, Manuel Calvopiña, Victoria Nipáz, Belén Vicente-Santiago, Julio López-Albán, Pedro Fernández-Soto, Ángel Guevara, Antonio Muro

**Affiliations:** 1 Universidad Central del EcuadorUniversidad Central del EcuadorCentro de Biomedicina, Carrera de MedicinaQuitoEcuadorUniversidad Central del Ecuador, Centro de Biomedicina, Carrera de Medicina, Quito, Ecuador; 2 Universidad de Las AmericasUniversidad de Las AméricasQuitoEcuadorUniversidad de Las Américas, Quito, Ecuador; 3 Universidad de SalamancaUniversidad de SalamancaFaculty of PharmacyTropical Disease Research CentreSalamancaSpainUniversidad de Salamanca, Faculty of Pharmacy, Tropical Disease Research Centre, Group e-INTRO, Salamanca, Spain

**Keywords:** *Amphimerus* spp, Ecuador, ELISA, diagnosis

## Abstract

**BACKGROUND:**

*Amphimerus* spp. is a liver fluke that infects humans and domestic animals. It is highly prevalent in some Ecuadorian communities. Currently, diagnosis is based on the microscopic observation of eggs in faeces, but this has variable sensitivity. More sensitive methods are needed for diagnostic testing.

**OBJECTIVE:**

The main objective of this work was to develop an enzyme-linked immunosorbent assay (ELISA) using crude antigens from *Amphimerus* spp. adult worms to detect anti-*Amphimerus* IgG in human sera.

**METHODS:**

Crude somatic antigens were obtained from adult *Amphimerus* spp. worms. Human sera from 119 patients were tested: 48 from individuals with a confirmed *Amphimerus* spp. infection, 78 from non-infected Ecuadorians living in the endemic region, 60 from persons living in non-endemic areas (20 Ecuadorians, 20 Europeans, and 20 Africans), and 33 who had other parasitic and non-parasitic infections.

**PRINCIPAL FINDINGS:**

Results were analysed using the receiver-operator characteristic (ROC) curve analysis with an area under curve (AUC) value of 0.967. The accuracy of the ELISA was high. The sensitivity was 85.0% [95% confidence interval (CI): 80.3-89.7%] and the specificity was 71.0% (95% CI: 65.2-76.8%). Some cross reactivity was detected against *Paragonimus mexicanus*, *Fasciola hepatica*, Schistosomiasis, *Taenia solium*, *Strongyloides stercoralis*, *Mansonella* spp*.,* and *Vampirolepis nana.*

**MAIN CONCLUSIONS:**

We have developed the first ELISA technique that detects anti-*Amphimerus* IgG in human sera with good sensitivity, repeatability and reproducibility. However, more specific antigens are needed to further enhance performance of this assay. Regardless, this ELISA test could be useful for early diagnosis and prompt treatment of human *Amphimerus* spp. infections.

Amphimeriasis is a zoonotic disease caused by infection with the liver fluke *Amphimerus* spp., a member of the *Opisthorchiidae* family that includes *Clonorchis sinensis*, *Opisthorchis viverrini* and *O. felineus.* The genus *Amphimerus* (Barker, 1911) infects several wild and domestic mammals in the Americas, and it has been reported in cats, dogs, marsupials, and rodents from Canada, the United States, Costa Rica, Panama, Colombia, Ecuador, Brazil and Peru (Artigas & Perez 1962, Thatcher 1970, Miyazaki et al. 1978, de Moraes Neto et al. 1998, Bowman 2002). These flukes infect humans after ingestion of raw or undercooked freshwater fish parasitised with viable metacercariae. Recently, *Amphimerus* spp. infections were found in 34% of an indigenous Chachi population living in the tropical rain forest of Northwestern Ecuador. Since the Chachi community habitually consumes smoked or lightly cooked freshwater fish, an estimated 20,000 people are at risk of acquiring this disease (Calvopiña et al. 2011). Furthermore, a recent study reported a very high prevalence of infection in domestic cats and dogs living in Chachi communities (Calvopiña et al. 2015). Further studies by the authors (MC & WC) found infected people in several other provinces of Ecuador (unpublished observations).

Adult parasites of the genus *Amphimerus* spp. grow and parasitise the host’s intra- and extra-hepatic bile ducts (Calvopiña et al. 2015). It is well documented that other members of the *Opisthorchiidae* are responsible for heavy and long-lasting infections that lead to hepatobiliary diseases including hepatomegaly, cholangitis, cholecystitis, and cholangiocarcinoma (Sripa et al. 2011). *Amphimerus* spp. infection may also occur in humans. However, it is mostly an asymptomatic disease, occasionally causing non-specific, generalised symptoms. To date, there are no comprehensive descriptions of this disease. However, histopathological studies in cats and a double-crested cormorant infected with *Amphimerus* spp. showed the presence of liver cirrhosis and pancreatitis (Rothenbacher & Lindquist 1963, Pense & Childs 1972).

At present, the diagnosis of *Amphimerus* spp. infections in humans is achieved by direct microscopic observation of eggs in the patient’s faeces. Observation of eggs after formalin-ether concentration has also been utilised. The formalin-ether method is used on samples from cats and dogs, but this technique is not routinely conducted in local laboratories in Ecuador (Calvopiña et al. 2015). The sensitivity of direct microscopic observation is up to ten times lower than the formalin-ether method (Calvopiña et al. 2011). Likewise, it has been demonstrated that the fluke eggs of other *Opisthorchiidae* parasites can be detected in the stools. This represents the best way to obtain a definitive diagnosis, although this approach becomes increasingly unreliable in cases of low-worm burden (Johansen et al. 2010). Moreover, human amphimeriasis is asymptomatic in most cases and does not show pathognomonic signs and symptoms. Therefore, physicians can easily miss *Amphimerus* spp. infections or have difficulties making a differential diagnosis in endemic areas and, even more so, in non-endemic areas where infected migrant people may require medical attention. A reliable diagnosis test is needed to ensure appropriate treatment and prevent chronicity to reduce the risk of developing liver damage.

Immunological techniques, such as antibody-based methods using enzyme-linked immunosorbent assay (ELISA), have shown high sensitivity and specificity for diagnosing various parasitic infections (Elkins et al. 1991, Guevara et al. 1995). Of particular note, ELISA was used to detect parasites of the *Opisthorchiidae* family, and this technique performed the best among all serological tests evaluated (Meniavtseva et al. 1996). *Clonorchis sinensis* and *Opisthorchis* spp. induce robust immune responses and significantly increase the levels of IgG in experimental animals, which is similar to observations in humans (Elkins et al. 1991, Gómez-Morales et al. 2013). The detection of specific antibodies has been considered a complementary tool to establish a definitive diagnosis for liver fluke infections (Upatham & Viyanant 2003). Antigen-based techniques using crude adult extracts have been used for immunodiagnosis of *O. viverrini* and *C. sinensis* infections, albeit with varying levels of sensitivity and specificity (Wongratanacheewin et al. 1988, 2003, Poopyruchpong et al. 1990, Sawangsoda et al. 2012).

For *Amphimerus* infections, there are no immunological diagnostic methods currently available. In the present study, we developed an immunological assay using total crude somatic extract antigens to detect anti-*Amphimerus* IgG antibodies in sera from infected people in Ecuador.

## MATERIALS AND METHODS

*Ethics statement* - The present study was approved by the Ethics Committee of the Universidad Central del Ecuador (License number LEC IORG 0001932, FWA 2482, IRB 2483, COBI-AMPHI-0064-11). Oral informed consent was obtained from all individuals participating in the study prior to the collection of biological samples for parasitological and immunological evaluation. Furthermore, written consent from animal owners was obtained prior to the euthanisation of cats that had been naturally infected with *Amphimerus* spp. to obtain adult parasites.

*Crude somatic extract of Amphimerus spp*. *and antigen preparation* - Adult *Amphimerus* spp. were obtained from cat livers following euthanasia by injection of a lethal dose of ketamine. The adult worms were washed with sterile phosphate-buffered saline (PBS) until the host’s blood was completely removed. Afterwards, clean liver flukes were suspended in sterile PBS at a concentration of 30 worms/mL and homogenised in a buffer [10 mM Tris base, 1 mM EDTA, 500 µL of a protease inhibitor cocktail (UltraCruz® Protease Inhibitor Cocktail Tablet, Santa Cruz Biotechnology, catalogue number sc - 29130)]. The suspension was frozen and thawed three times and sonicated three times at 70 kHz for one minute each cycle using a sonicator (Vibra-Cell, Sonics and Materials Inc., Danbury, CT, USA). The samples were then centrifuged at 16000 *g* for 30 min at 4ºC. The crude antigen was lyophilised and stored at 4ºC until used. The protein concentration of the antigen was determined using the commercial micro-BCA™ Protein Assay Kit (Thermo Scientific Pierce, Waltham, MA, USA).

*Human sera* - A total of 219 human serum samples were used to test the diagnostic value of the crude antigen by ELISA. Of these, 48 sera samples were from patients who had received a definitive diagnosis, which was demonstrated microscopically by the presence of *Amphimerus* spp. eggs in their stools [true positive (TP)]. Additionally, 60 sera samples were from people living in non-endemic areas, including 20 Ecuadorians, 20 Europeans, and 20 Africans, who were free of trematode infections [true negative (TN)]. A group of 78 serum samples were obtained from people living in endemic areas, all of whom tested negative for a *Amphimerus* spp. infection based upon a coprologic test. Finally, 33 serum samples were derived from patients infected by other parasites or viruses including *Paragonimus mexicanus* (2), *Fasciola hepatica* (3), *Schistosoma mansoni* (2), *Schistosoma haematobium* (2), *Echinococcus granulosus* (2), *Mansonella* spp*.* (2), hookworms (2), *Vampirolepis nana* (2), *Strongyloides stercoralis* (2), *Taenia solium* (1), *Ascaris lumbricoides* (2), *Onchocerca volvulus* (1), *Leishmania* spp. (1), *Giardia intestinalis* (2), *Chilomastix mesnili* (1), *Blastocystis hominis* (1), *Microsporidium parvum* (1), *Plasmodium falciparum* (1), *Dientamoeba fragilis* (1), and hepatitis B virus (2). Of all the serum samples used in this study, 81% were from adults (14-98 years old), and approximately 45% were from females.

Serum samples from Europeans and Africans, as well as individuals with other parasitic and viral infections, were provided by IBSAL-CIETUS. The diagnoses of parasitic infections were based on parasitological and/or serological tests. Sera from Ecuadorians were obtained by venipuncture using disposable needles and 10 mL vacuum tubes (VACUETTE^®^ Bio-one GmbH, Austria). These samples were obtained between January and May 2015. Blood was allowed to clot, and was subsequently centrifuged for 15 min at 1000 *g.* Serum was then aliquoted in cryovials and stored at -20ºC until use. All participants provided oral informed consent before blood samples were collected.

*ELISA* - A standard ELISA protocol was developed to test all human serum samples. Briefly, 96-well microtiter plates (Sigma, St. Louis, MO, USA) were coated with 100 µL/well of 4 µg/mL *Amphimerus* spp. crude extract antigens from adults. The crude extract had been reconstituted in carbonate buffer (pH 9.6). The plates were incubated overnight at 4ºC. Next, the plates were washed three times with PBS (pH 7.3) containing 0.05% Tween-20 (PBS-T20) (Sigma). Plates were then blocked in 200 µL/well of PBS with 0.5% BSA and 1% Tween-20 at 37ºC for 1 h. After additional washing steps, 100 µL of sera diluted 1:50 in PBS-T-20 was added to each well in duplicate, and plates were incubated at 37ºC for 1 h. After another wash step, 100 µL/well of 1:1000 diluted goat anti-human IgG peroxidase-labelled antibody (Sigma) was added and incubated at 37ºC for 1 h. Finally, another wash was performed and 100 µL/well of 5.3 µg/mL O-phenylene-diamine (OPD) and 8 µL of hydrogen peroxide in citric acid buffer were added. Plates were incubated at room temperature for 15 min, and the reaction was stopped by adding 50 µL/well of 1N H_2_SO_4_ solution. Optical density (OD) values were obtained by reading the plates at 492 nm using an ELISA plate microtitre reader (Ear400FT ELISA reader Lab. Instruments). All samples were tested in duplicate, and the OD mean was calculated.

*Statistical analysis* - A serological index (SI) was calculated for each OD, and this was used to establish a cut-off value for the ELISA using the following formula: SI = [(PS-NC) / (PC-NC)] X 100, where NC and PC are the negative and positive controls, respectively, and PS is the problem sample (Hernández-González et al. 2008).

Receiver-operator characteristic (ROC) curve analysis was used to determine the diagnostic value. The ROC curve is a graphical plot of the sensitivity versus “1 - specificity” for a binary classifier system. Using various cut-off values, this allows the selection of the cut-off value that gives the best balance of sensitivity and specificity for the test under consideration (Gómez-Morales et al. 2013). In order to determine which cut-off provided the most accurate result, we used the mean OD of the duplicates and the SI to calculate the area under the ROC curve for each cut-off. The specificity and sensitivity were interpreted according to the ROC analysis. The SI and OD in the different groups were expressed as the mean and standard error of the mean (SEM). Positive and negative predictive values were calculated, as described elsewhere (Fernández & Días 2003).

All statistical calculations were performed using SPSS (version 22.0), which is available from: https://www.ibm.com.

## RESULTS

**Fig. 1:**
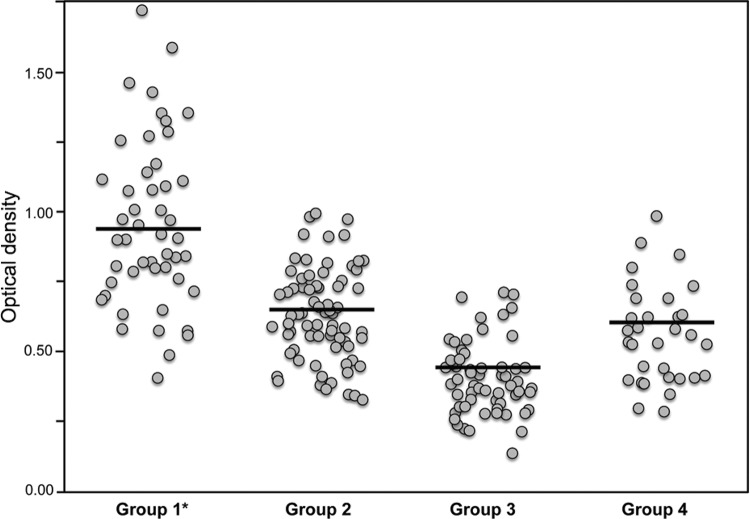
: values of optical density determined for the *Amphimerus* spp. crude antigen determined by enzyme-linked immunosorbent assay (ELISA) using sera from patients. We included sera from patients diagnosed with amphimeriasis based upon the detection of eggs (group 1), sera from healthy people living in amphimeriasis-affected areas (group 2), sera from healthy people living in areas unaffected by amphimeriasis (group 3), and sera from patients suffering from unrelated helminthic, protozoan, or viral infections (group 4). The horizontal line represents the mean values. The asterisk represents significant differences in the mean between group 1 and the other groups.

**Fig. 2:**
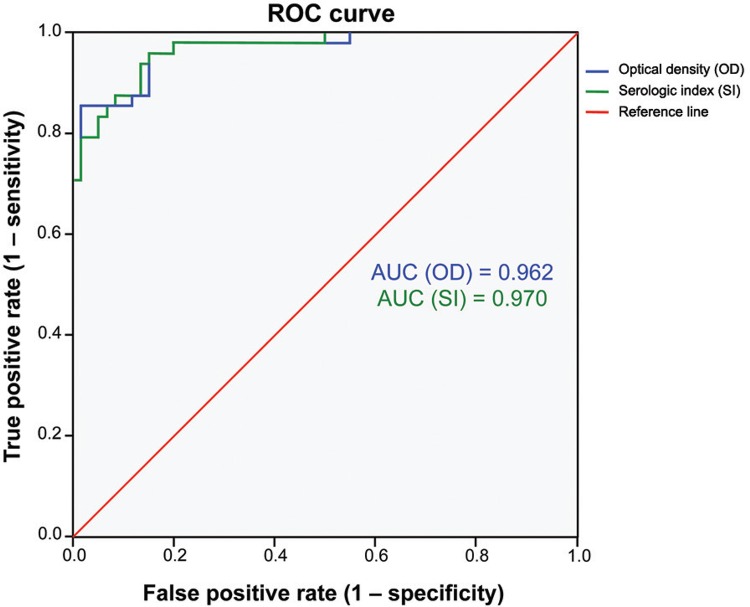
: the receiver-operator characteristic (ROC) curve. The ROC curve was generated using data from 48 sera samples from people infected with *Amphimerus* spp. and 60 sera samples from healthy people residing in non-endemic areas. The area under the curve (accuracy) for the serologic index (SI) was 0.970. For the optical density (OD) was 0.962.

ROC curves were built with data from two defined true positive and true negative reference populations. The ODs of the 219 sera analysed are presented in Fig. 1. The mean OD ± standard deviation (SD) for the group of patients with confirmed amphimeriasis was the highest (0.938 ± 0.304) of those calculated. The remaining groups presented low means: ODs: 0.627 ± 0.144 for healthy patients from zones endemic for amphimeriasis, 0.412 ± 0.120 for healthy people from non-endemic areas, and 0.566 ± 0.206 for patients with other helminths, protozoan, and viral infections. Comparative analysis of the mean ODs detected significant differences between values for group 1 and those for the remaining groups (p < 0.0001). The area under curve was 0.967 for the mean OD value of the duplicates and 0.970 for the SI, indicating that the two parameters provided equally accurate results (Fig. 2). The ROC optimised cut-off was 18% for SI and 0.624 for the OD values; on the basis of these cut-off values, the sensitivity reached 85% (95% CI 78.3% to 91.7%) and the specificity was assessed as 94% (95% CI: 89.5% to 98.5%).

The diagnostic sensitivity and specificity of the ELISA in this study, including serum samples from people who lived in endemic areas and people with other parasitic and non-parasitic infections, are summarised in Table. The sensitivity of the test was 85% (95% CI: 80.3% to 89.7%), but the specificity dropped to 71% (95% CI: 65.2% to 76.8%) with a positive predictive value of 46% (95% CI: 39.4% to 52.6%) and negative predictive value of 86.5 (95% CI: 84.2% to 88.8%). For this reason, we eliminated 78 healthy people from areas where amphimeriasis is endemic.

**TABLE:** Diagnostic sensitivity, specificity, and positive and negative predictive values [cut-off 18%, 95% confidence interval (CI)] for serum samples from people diagnosed with *Amphimerus* spp. infection. The infection was confirmed by detection of *Amphimerus* eggs in stool samples (infected). Serum samples from people living in endemic areas who were negative for the presence of *Amphimerus* eggs in stool and serum samples from people suffering from other parasitic or non-parasitic infections (uninfected) were also examined

*Amphimerus* spp. infection					
ELISA result	Infected	n	Uninfected	n	Total
Positive	TP	41	FP	48	89
Negative	FN	7	TN	123*	130

Total		48		171	219

Sensitivity: = 85.0% (95% CI: 80.3 - 89.7%), it was calculated as TP/(TP+FN); specificity: = 71.0% (95% CI: 65.2 - 76.8%), it was calculated as TN/(TN+FP); positive predictive value = 46.0% (95% CI: 39.4 - 52.6%), it was calculated as TP/(TP+FP); negative predictive value = 86.5 (95% CI: 84.2% to 88.8%), it was calculated as TN/(TN+FN); ELISA: enzyme-linked immunosorbent assay; FN: false negative; FP: false positive; TN: true negative; TP: true positive; *: for this value we eliminated 78 healthy people from endemic amphimeriasis area.

Seven serum samples (17%) yielded false-negative results, and 48 samples (28%) yielded false-positive results. Of these false-positives, 34 were from people who live in endemic areas, and 14 were from persons with other parasitic infections (two were infected with *P. mexicanus*, three with *S. mansoni*, and one for each of these parasites: *T. solium*, *S. stercoralis*, *F. hepática*, *Mansonella* spp., and *V. nana*).

## DISCUSSION

The present study shows for the first time that the *Amphimerus* spp. liver fluke infection induces an immune response in human hosts. This response is characterised by the production of IgG antibodies, which can be detected by ELISA using crude somatic extract antigens. Our data demonstrate that the ELISA has good sensitivity for sera from patients confirmed to be infected with this parasite.

Human amphimeriasis is an emerging food-borne trematode infection commonly found in tropical areas of Ecuador. It has a high prevalence in the indigenous Chachi population of Ecuador (Calvopiña et al. 2011). Our recent unpublished findings demonstrate that people in other communities along the Pacific coast of Ecuador are also infected with *Amphimerus* spp. In those studies, direct egg detection via faecal examination by microscopy was performed. However, as reported before, the formalin-ether concentration technique was more sensitive than a direct smear (Calvopiña et al. 2011). For *Opistorchiidae* family members, many of these trematodes have morphologically indistinguishable eggs. This includes minute intestinal trematodes, such as *Haplorchis* spp., *Echinostoma* spp., *Metorchis* spp., and *Metagonimus* spp. As a result, the low specificity of egg identification during common low-grade infections is a major problem for diagnostic methods (Johansen et al. 2010). Several studies for other *Opisthorchiidae* flukes such as *C. sinensis* and *O. viverrini*, have demonstrated that crude antigens work well for inducing a B-immune response during human infections (Wongratanacheewin et al. 1988, Meniavtseva et al. 1996). Thus, more sensitive and versatile serodiagnostic tests based on ELISAs have become the predominant assays used in biomedical research to diagnose clonorchiasis, opistorchiasis, paragonimiasis, and fasciolosis.

To the best of our knowledge, no serological tests have been developed and validated for amphimeriasis using crude antigens. This was our rational for evaluating ELISAs against a large panel of sera from individuals confirmed to have a *Amphimerus* spp. infection; people living in endemics area who are negative for eggs; people infected with viruses, protozoa, and other helminths; and, lastly, healthy people.

Regarding sensitivity, we found that our ELISA detected 85.0% (95% CI: 80.3-89.7%) of the TP samples. Our results are in accordance with those obtained for other parasites of the *Opisthorchiidae* family using crude antigens. For instance, the sensitivity of a previously reported ELISA for *C. sinensis* infection was 88.2% (Choi et al. 2003), and for *O. viverrini* infection, the range was between 83.3% and 100% (Poopyruchpong et al. 1990). This moderate sensitivity could be explained by level of worm burden, as the serum IgG levels against *O. viverrini* correlated with the overall egg count (Elkins et al. 1991, Wongratanacheewin et al. 2003). We propose that the sensitivity could also be affected by free-living parasites in the bile ducts.

In our results, 34 (19.8%) serum samples were not identified as positive for an infection by microscopic examination. However, these samples were correctly scored as positive sera by ELISA. This considerable improvement in the identification of seropositive cases for *Amphimerus* spp. in egg-negative individuals is probably due to the presence of a paucisymptomatic disease or a delayed diagnosis, which is common in *Opisthorchis* spp. infections (Armignacco et al. 2008). This suggests that this ELISA will be able to positively diagnose more infected people than faecal examination by microscopy can. In the ELISA, more than 95% of the differences between OD values of serum duplicates were less than two SDs, indicating the high reproducibility of the ELISA.

On the other hand, the specificity of the ELISA dropped to 71% (95% CI: 65.2% to 76.8%) when sera from people living in endemic areas or from those with other health disorders unrelated to *Amphimerus* spp. were tested. Several authors have hypothesised that the main drawback of serologically based diagnostic methods that detect circulating antibodies, especially for infections caused by helminths, is the cross-reactivity that can occur when testing crude extracts containing parasites (Hong 1988, Sirisinha et al. 1990). This is particularly important in developing countries where people could be infected with several species of liver and intestinal flukes, in addition to other helminths (Sawangsoda et al. 2012). The relatively low specificity we observed was similar to results obtained in comparable studies of infections of *C. sinensis* and *Opisthorchis* spp. In South Korea, the ELISA for *C. sinensis* yielded an extremely low specificity of 33.3% (Kim et al. 2010). However, in another study, the specificity improved to 87.8% (38) and 81% for *O. viverrini* (Sripa et al. 2012).

Importantly, the specificity of our assay was assessed using heterologous sera from 33 patients infected with various other parasites. This included 23 helminths and eight protozoa, such as the liver fluke *F. hepatica*, the lung fluke *P. mexicanus*, and the blood flukes *S. haematobium* and *S. mansoni*. Sera from all of these pathogen-infected patients yielded false-positive results that impacted the overall specificity of the test, indicating that the use of somatic antigens in the diagnosis of amphimeriasis in areas where paragonimiasis is endemic produces many false-positive results. Of note, however, only paragonimiasis in Ecuador occurs in endemic areas where sera were collected (Calvopiña et al. 2014). Another important limitation of the current study is that we did not evaluate sera from patients infected with other *Opistorchiidae* family members, *C. sinensis* and *Opisthorchis* spp. In Ecuador, where amphimeriasis is currently prevalent, there are no reported cases of clonorchiais, opistorchiasis, or infections with another minute intestinal trematodes that occurs mainly in Asian countries (Johansen et al. 2010, Calvopiña et al. 2011). Thus, this ELISA method may be sufficient to accurately diagnose infections of *Amphimerus* spp. in endemic regions of Ecuador.

In summary, we have developed an ELISA that would be useful for epidemiological surveys in areas where *Amphimerus* spp. is endemic. However, further attempts to refine the assay are needed. This includes obtaining antigens that are more specific for *Amphimerus* spp., such as the excretory/secretory antigens that have provided more specificity than somatic soluble extracts for other trematode detection methods (Gómez-Morales et al. 2013). Regardless, our test shows good overall performance for the diagnosis of the *Amphimerus* infection as defined by sensitivity, repeatability, and reproducibility. We conclude that this method is useful for detecting anti-*Amphimerus* antibodies in human sera, facilitating early diagnosis and prompt treatment.
